# Ginkgo Biloba Leaf Extract Attenuates Atherosclerosis in Streptozotocin-Induced Diabetic ApoE-/- Mice by Inhibiting Endoplasmic Reticulum Stress via Restoration of Autophagy through the mTOR Signaling Pathway

**DOI:** 10.1155/2019/8134678

**Published:** 2019-03-18

**Authors:** Jinfan Tian, Mohammad Sharif Popal, Yanfei Liu, Rui Gao, Shuzheng Lyu, Keji Chen, Yue Liu

**Affiliations:** ^1^Department of Cardiology, Beijing Anzhen Hospital, Capital Medical University, Beijing Institute of Heart, Lung and Blood Vessel Diseases, Beijing 100029, China; ^2^Cardiovascular Disease Center, Xiyuan Hospital, China Academy of Chinese Medical Sciences, Beijing 100091, China; ^3^Graduate School, Beijing University of Chinese Medicine, Beijing 100029, China; ^4^Institute of Clinical Pharmacology of Xiyuan Hospital, China Academy of Chinese Medical Sciences, Beijing 100091, China

## Abstract

**Background:**

There is a crosstalk between endoplasmic reticulum stress (ERS) and autophagy, and autophagy could attenuate endoplasmic reticulum stress-mediated apoptosis. Ginkgo biloba leaf extract (GBE) exerts vascular protection functions. The purpose of the present study is to investigate the role of autophagy in diabetic atherosclerosis (AS) and the effect of GBE on autophagy and ERS.

**Methods:**

Network pharmacology was utilized to predict the targets and pathways of the active chemical compounds of Gingko biloba leaf to attenuate AS. ApoE^−/−^ mice were rendered diabetic by intraperitoneal ingestion with streptozotocin combined with a high-fat diet. The diabetic mice were divided into five groups: model group, atorvastatin group, rapamycin group, and low- and high-dose GBE groups. Serum and tissue markers of autophagy or ERS markers, including the protein expression, were examined.

**Results:**

The mammalian target of rapamycin (mTOR) and NF-*κ*B signaling pathways were targeted by the active chemical compounds of GBE to attenuate AS predicted by network pharmacology. GBE reduced the plaque area/lumen area and the plaque lipid deposition area/intimal area and inhibited the expressions of CD68, MMP2, and MMP9. Rapamycin and GBE inhibited the expression of mTOR and SQSTM1/p62 which increased in the aorta of diabetic mice. In addition, GBE reduced the expression of ERS markers in diabetic mice. GBE reduced the serum lipid metabolism levels, blood glucose, and inflammatory cytokines.

**Conclusion:**

Impaired autophagy and overactive endoplasmic reticulum stress contributed to diabetic atherosclerosis. mTOR inhibitor rapamycin and GBE attenuated diabetic atherosclerosis by inhibiting ERS via restoration of autophagy through inhibition of mTOR.

## 1. Introduction

Atherosclerosis as one of the vascular complications of diabetes mellitus is accelerated by oxidative stress and endoplasmic reticulum stress. Therefore, amelioration of endoplasmic reticulum stress-mediated apoptosis is significant for the attenuation of diabetic atherosclerosis. Three pathways of endoplasmic reticulum stress, including IRE1-XBP1, PERK-eIF2*α*, and IRE1-JNK, contribute to activation of autophagy [1]. Autophagy is a nonapoptotic cell death manner, without increasing the inflammation of plaques. Furthermore, autophagy attenuates apoptosis by degrading unfolded proteins and impaired cell organelles. The protective function of macrophage autophagy involves the regulation of cholesterol metabolism [2]. Free cholesterol was hydrolyzed from cholesteryl esters by autophagy lysosomes in foam cells, and its efflux was mediated by ATP-binding cassette transport protein 1. The impaired macrophage autophagy results in decreased free cholesterol efflux and accumulation of apoptotic foam cells attributable to lipid overload, leading to the increased lipid content in the plaques, increased necrotic core, and secondary inflammatory reaction, which consequently promote the vulnerability of atherosclerotic plaques [3]. In the models of atherosclerotic ApoE-/- mice, high-fat diet inhibits Beclin1-mediated protective effects of macrophage autophagy, thus accelerating the progression of atherosclerosis [4]. Rapamycin has the ability to restore impaired autophagy by inhibiting the mammalian target of rapamycin (mTOR), leading to selective clearing of macrophages, increased cholesterol efflux, reduction of apoptotic cells in the plaques, and stabilization of atherosclerosis in ApoE-/- mice [5–7].

There is a crosstalk between endoplasmic reticulum stress and autophagy, and autophagy protectively inhibits endoplasmic reticulum stress-mediated apoptosis in a negative feedback manner. According to Liao et al. [2], autophagy inhibits oxidative stress and endoplasmic reticulum stress-mediated macrophage apoptosis. Glomerular autophagy has the ability to attenuate endoplasmic reticulum stress and cell injury in mesangial cells induced by advanced glycation end products (AGE) [8]. Autophagy exerts a protective role in AGE-induced early injury of human vascular endothelia cells, featured by restoration of LC3II [9]. 7-Ketone-induced autophagy attenuates atherosclerosis by inhibition of endoplasmic reticulum stress [10]. On the other hand, the excess of autophagy also contributes to atherosclerosis as a form of cell death, indicating moderate autophagy protecting against atherosclerosis. Phosphatidylinositol 3 kinase- (PI3K-) Akt-mTOR and AMP-activated protein kinase- (AMPK-) mTOR are the two major pathways regulating autophagy. Although it has been well established that rapamycin is protective in atherosclerosis by inducing autophagy via inhibition of mTOR, the role of mTOR in diabetes mellitus is complex. Activation of mTOR promotes the secretion of insulin and increases insulin sensitivity; on the contrary, mTOR may lead to glucose intolerance through blocking of the insulin receptor substrate 1 (IRS1) by phosphorylation of p70S6K in a negative feedback loop manner [11, 12]. Hyperleptinemia can coexist with diabetes mellitus and has the ability to stimulate the activity of mTOR and promote the vascular smooth muscle cell proliferation [12]. The mTOR pathway was activated in the liver and skeletal muscle of obese rats involved in obesity-induced insulin resistance [13]. Autophagy is essential to islet function and survival; on the contrary, inadequate autophagy can lead to islet degeneration and reduced insulin secretion [14, 15]. According to den Hartigh et al., low-grade chronic mTORC1 inhibition improved insulin sensitivity attributable to signaling through reduced phosphorylated p70S6K, which might be beneficial in antiobesity and anticardiovascular disease therapies [16]. AMPK, upstream of mTOR, exerts cardiovascular protective function by inhibiting mTOR [17]. However, this protective effect attenuates in the context of high glucose due to inactivation of AMPK [18]. In the condition of diabetes, the cardio SIRT1 is a molecule directly or indirectly linked to the mTOR signaling pathway and autophagy. SIRT1 prevents endothelial senescence induced by hyperglycemia and ox-LDL through upregulation of autophagy via activation of AMPK [19, 20]. SIRT1 inhibition promotes atherosclerosis through impaired autophagy [21]. SIRT1 overexpression in neurons promotes neurite outgrowth and cell survival [22] and blocks the senescence of mesangial cells induced by high glucose through inhibition of mTOR signaling [23]. According to Hou et al. [24], inhibition of mTOR pathways prevents high glucose-induced inhibition of autophagy and cardiomyocyte injury. Consequently, it is hypothesized that the overactivated mTOR increases insulin resistance and the risk of cardiovascular diseases. However, there is lack of study regarding the activity of mTOR signaling pathways in diabetic atherosclerosis.

The impairment of the initiation stage of autophagy is characterized by decreased LC3II and increased SQSTM1/p62. Failure of the terminal phases of autophagy is characterized by increased SQSTM1/p62 in the cell, indicating an inability to clear autophagosomes and degrade p62 [25]. The increased expression and bioactivity of SQSTM1/p62 are closely associated with atherosclerosis [26]. The high concentration of ox-LDL blocked autophagic flux, leading to impairment function of autophagic degradation and SQSTM1/p62 aggregation. The accumulation of SQSTM1/p62 is involved in the increased MMP9 expression mediated by the NF-*κ*B pathway, resulting in instability of the atherosclerotic plaques [27]. According to Fetterman et al. [25], inadequate autophagy featured by increased p62 promotes endothelial dysfunction in diabetic patients. Zhang et al. [28] revealed that high glucose inhibits autophagy in cardiac microvascular endothelial cells (CMECs), featured by activation of the mTOR pathway and increased SQSTM1/p62 levels, enhancing the apoptosis of CMECs. On the contrary, rapamycin inhibits high glucose-induced CMEC apoptosis by inhibiting mTOR signaling and through degrading SQSTM1/p62. The expressions of these hallmarks of autophagy in the context of diabetic atherosclerosis are still unknown.

Recently, accumulated studies reported that herbals could protect against ischemic cardiomyopathy by regulating autophagy [29]. Gingko biloba leaf extract (GBE), which contains terpenoids, flavonoids, alkylphenols, polyprenols, and organic acids, has long been used for the treatment of cardiovascular disease [30]. The mechanism by which GBE exerts vascular protection effects involves anti-inflammation, antioxidant, and antiplatelet. Ginkgo biloba K has been shown to attenuate endoplasmic reticulum stress and cell apoptosis in the infarct myocardium through upregulation of autophagy by inducing XBP1 [1]. GBE protects against myocardial ischemic/reperfusion injury in the context of high glucose by the mechanism of antioxidants [31]. According to our previous study, GBE attenuated streptozotocin- (STZ-) induced diabetic ApoE-/- mouse injury by attenuation of endoplasmic reticulum stress [32]. In vitro, GBE inhibited the adhesion of macrophages to endothelial cells induced by high glucose through reduction of the expression of IL-6 [33]. Currently, there are few studies regarding the effect of GBE on diabetic atherosclerosis in vivo. Furthermore, it is still unclear if GBE has the ability to attenuate diabetic atherosclerosis through regulating the interaction between autophagy and endoplasmic reticulum stress. The present study is aimed at investigating autophagy and endoplasmic reticulum stress activity in diabetic atherosclerosis and whether GBE ameliorates diabetic atherosclerosis by inhibiting endoplasmic reticulum via upregulation of autophagy.

## 2. Materials and Methods

### 2.1. Prediction Analysis of Pharmacological Mechanism Based on Network Pharmacology

**Figure 1:**
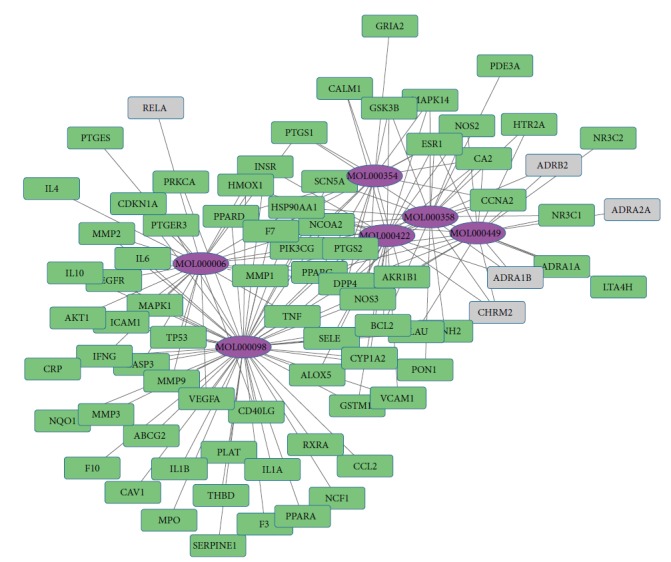
Compound-target network. The purple nodes indicate the 6 active chemical compounds with degrees from 58 to 19. The other nodes indicate corresponding targets for atherosclerosis. The lines indicate the interaction between these chemical active compounds and targets.

**Figure 2:**
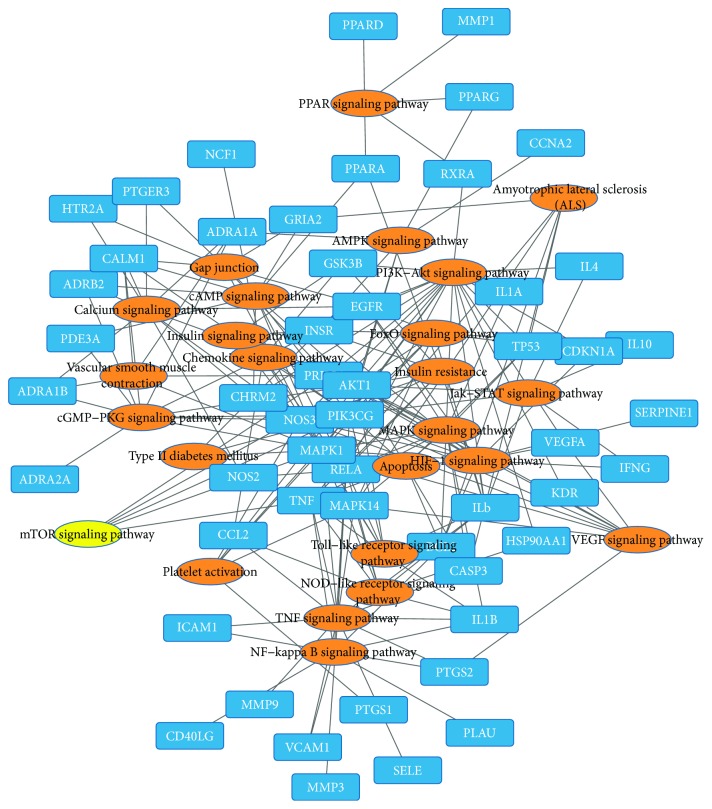
Target-pathway network. The blue nodes indicate 79 targets for atherosclerosis. The orange nodes indicate the corresponding pathways obtained in the DAVID database, and the yellow node indicates mTOR signaling. The lines indicate the interaction between these targets and signaling pathways.

**Figure 3:**
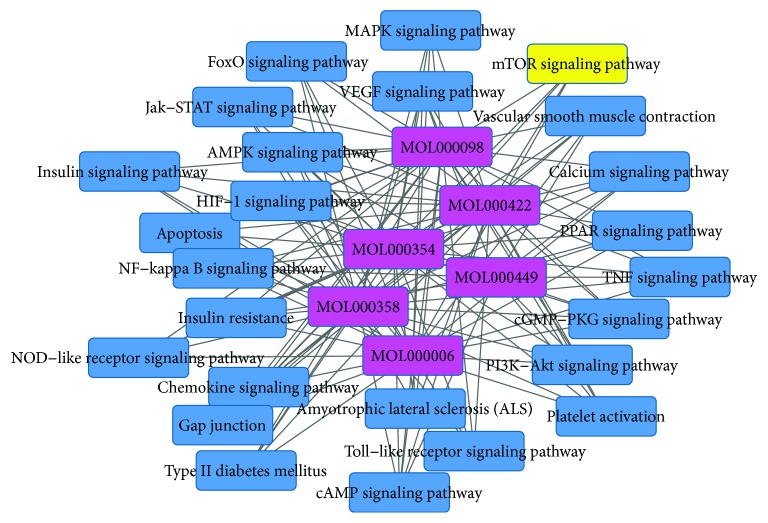
Compound-pathway network. The purple nodes indicate the 6 active chemical compounds. Blue and yellow nodes indicate signaling pathways corresponding to these active chemical compounds. The lines indicate the interaction between compound and signaling pathways.

Gingko biloba leaf contains terpenoids (including ginkgolides and bilobalide), flavonoids (primarily quercetin, kaempferol, isorhamnetin, luteolin, rutin, apigenin, and myricetin), alkylphenols, polyprenols, and organic acids. 223 chemical compounds were obtained from the TCMSP database (http://lsp.nwsuaf.edu.cn/tcmsp.php). A bioavailability (OB) >30%, drug-like >0.18, was set as the threshold for further extraction and optimization of the ingredients in ADEM, and 25 active chemical compounds were used for further analysis [34]. 227 targets for the 25 active chemical compounds were obtained in the TCMSP database. 348 targets for atherosclerosis were achieved in the DrugBank database (http://www.drugbank.ca/), TTD database (http://bidd.nus.edu.sg/BIDD-Databases/TTD/TTD.asp), GAD database, and available literature. The gene symbols were retrieved in the UniProtKB database (http://www.uniprot.org) using protein names. 81 targets were subsequently achieved by intersection of targets for active chemical compounds and targets for atherosclerosis. Then, 6 active chemical compounds with degrees from 58 to 19 (quercetin, luteolin, kaempferol, beta-sitosterol, isorhamnetin, and stigmasterol) and 79 corresponding targets were used for further analysis. Inflammation cytokines IL-1*β*, IL-6, TNF, and MMP were included in these targets. The compound-target network was constructed by Cytoscape software (Figure 1). DAVID database (https://david.ncifcrf.gov/) was applied for KEGG pathway analysis, and 25 pathways for these 6 active compounds were achieved. The target-pathway network and compound-pathway network were constructed by utilizing Cytoscape software (Figures 2 and 3). These 6 active chemical compound target pathways include mTOR signaling pathways and NF-*κ*B-mediated inflammation signaling.

### 2.2. Reagents

GBE powder was purchased from Beijing Handian Pharmaceutical Co. Ltd., atorvastatin and rapamycin were purchased from Pfizer Pharmaceuticals Co. Ltd., and streptozotocin (STZ) was purchased from Sigma Co. Ltd. Ginkgo flavonoid and terpenoid contents in GBE in the present are 44.9% and 6.3%, respectively, whereas the amount of ginkgo acid is limited to <1 ppm.

### 2.3. Experimental Protocol

Male ApoE-/- mice, aged 6-7 weeks (weighing 19-21 g (C57BL/6J background, introduced from Jackson Laboratory of USA by Peking University Health Science Center Laboratory Animal Science Department; quality certification number SCXK (Beijing) 2016-0012)), were rendered diabetic by intraperitoneal injection of 50 mg/kg/day STZ diluted with citrate buffer (pH 4.5; final concentration, 1%) for 5 consecutive days [35], after rearing with high-fat diet for 4 weeks. The mice with plasma glucose level > 12 mmol/L served as models [35, 36]. The mice that received normal saline water served as model controls (*n* = 10). 20 C57BL/6J mice fed with chow diet served as normal controls. Diabetic mice were divided into five groups, namely, model group (normal saline water i.g., *n* = 16), atorvastatin group (10 mg/kg/day i.g., *n* = 16) [37], rapamycin group (1 mg/kg/day i.g., *n* = 16) [38], low-dose GBE group (200 mg/kg/day i.g., *n* = 18), and high-dose GBE group (400 mg/kg/day i.g., *n* = 18). The dose of GBE in the present study was based on a previous study [32, 39, 40]. The intragastric administration was performed one week after STZ injection. Atorvastatin, rapamycin, and GBE were dissolved and delivered by normal saline water. *Considering the less toxicity of normal saline compared to organic solvent when administered to mice, normal saline is a feasible choice for intragastric administration. To avoid precipitation, the drugs were blended before medication.*

**Figure 4:**
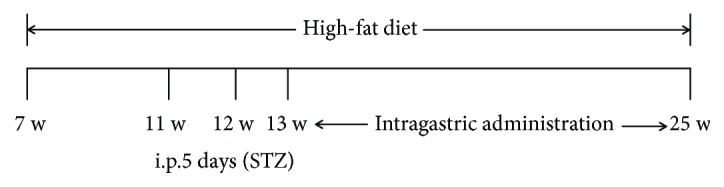
Experimental protocol.

All the mice were sacrificed after another 12-week gavage plus high-fat diet. At the end of the study course, there were 11 mice that survived in the model group; 8 mice in the model control group survived. In the atorvastatin group and rapamycin group, there were 14 and 15 survivors, respectively; the survivor mice in the low-dose GBE and high-dose GBE groups were 16 and 17, respectively. All the 20 mice in the normal group survived (see Figure 4). The experimental protocol was approved by the Institutional Animal Care and Use Committee of Xiyuan Hospital, China Academy of Chinese Medical Sciences, in accordance with the Guide for the Care and Use of Laboratory Animals published by the US National Institutes of Health.

### 2.4. Body Weight and Plasma Glucose Changes

The body weight and fasting plasma glucose level were recorded before the initial treatment of GBE and every four weeks thereafter. A glucometer (Roche) was used to determine the plasma glucose levels by the cutting tail method.

### 2.5. Tissue Preparation and Histological and Immunohistochemical Measurement

The chests were opened after the mice were anesthetized. The heart was perfused with heparin saline, and the hearts containing aortic root, together with the aortic arch to the iliac artery bifurcation, were harvested under aseptic conditions and fixed in 4% polyformaldehyde. The full length of the aorta is used for en face analysis by red “O” staining. The cross sections of the aortic sinus fixed in 4% paraformaldehyde and embedded in paraffin were used for H&E staining, MASSON staining, and immunohistochemical staining. Immunohistochemical staining was performed as the following: for *α*-SMA (rabbit polyclonal to *α*-SMA antibody; ab5694; 1 : 50), CD68 (rat monoclonal to CD68 antibody; ab53444; 1 : 50), MMP2 (rabbit polyclonal to MMP2 antibody; ab97779; 1 : 400), MMP9 (rabbit polyclonal to MMP9 antibody; ab38898; 1 : 100), mTOR (rabbit polyclonal to mTOR antibody; ab2732; 1 : 2000), Beclin1 (rabbit polyclonal to Beclin1; ab62557; 1 : 300), LC3B (rabbit polyclonal to LC3B; ab48394; 1 : 1000), and SQSTM1/p62 (rabbit polyclonal to SQSTM1/p62; ab91526; 1 : 100). Immunohistochemical semiquantitative analysis was conducted by an automated image analysis system (Image-Pro Plus 6.0; Media Cybernetics, MD Rockville, USA). The positive expressions of these indexes were represented by integral optical density (IOD).

### 2.6. Western Blot Analysis

The aortic tissues were snap-frozen in liquid nitrogen, weighed, homogenized, and resuspended in ice-cold lysis buffer. Protein concentration was determined with the bicinchoninic acid method. Equal amounts of protein from each sample were analyzed by sodium dodecyl sulfate-polyacrylamide gel electrophoresis (SDS-PAGE) and transferred onto a nitrocellulose membrane. Nonspecific sites were blocked by incubating the membranes with 5% nonfat milk and 0.2% Tween 20 in Tris-buffered saline for 2 h at room temperature. Primary antibodies incubated overnight at 4°C are as follows: mTOR (ab2732; 1 : 1000), Beclin1 (ab62557; 1 : 1000), LC3B (ab48394; 1 : 2000), SQSTM1/p62 (ab91526; 1 : 2000), CHOP (CST 2895S; 1 : 2000), p-JNK (CST 9251S; 1 : 1000), JNK (CST 9252S; 1 : 1000), and Caspase-12 (CST 2202S; 1 : 1000). Glyceraldehyde 3-phosphate dehydrogenase (GAPDH) was used as internal reference. The expressions of amTOR, Beclin1, LC3B, SQSTM1/p62, CHOP, and Caspase-12 were adjusted for GAPDH. The expression levels of p-JNK were adjusted for total JNK.

### 2.7. Blood Biomarker Detection

At the end of the 12-week period, fasting serum glucose and lipid profiles, including high-density lipoprotein cholesterol (HDL-c), total cholesterol (TC), triglycerides (TG), and low-density lipoprotein cholesterol (LDL-c) levels, were determined by an automated system before the mice were sacrificed. Blood samples were collected and centrifuged at 3000 rpm for 10 min.

Serum levels of inflammatory cytokines (IL-1, IL-6, IL-1*β*, TNF-*α*, and iNOS) were detected using commercially available ELISA kits, purchased from Beijing Fang Cheng Jia Hong Technology Co. Ltd. Five serial dilutions of the standard were prepared according to the manufacturer's instructions. Blank and sample wells were set, respectively. Sample diluent (40 *μ*L) was added to the sample wells in the precoated ELISA plates, followed by addition of the samples (10 *μ*L). After sealing the plates with a closure plate membrane, they were incubated for 30 min at 37°C. HRP-conjugated reagent (50 *μ*L) was added to all wells, except for the blank well. After incubation at 37°C, the liquid in the wells was removed, and the plate was washed with a wash liquid. Chromogen solution A (50 *μ*L) and chromogen solution B (50 *μ*L) were added to each well. The plates were incubated in the dark at 37°C for 15 min. The blank well was considered zero, and the absorbance of each well was measured at 450 nm within 15 min after adding the stop solution.

### 2.8. Statistical Analysis

SPSS 14.0 was used for data analysis. The measurement data were presented using means ± standard deviation (x ± s). One-way analysis of variance (ANOVA) was applied for comparing means among the groups. The least significant difference (LSD) test was used to multiple comparisons between the model group and the other groups. *P* < 0.05 was considered statistically significant. GraphPad Prism 5.0 software was used for graphical presentation.

## 3. Results

### 3.1. The Effects of GBE on Body Weight and Plasma Glucose Levels

The body weight was significantly reduced in diabetic mice compared to the model control group after the intraperitoneal injection of STZ for 1 week (22.3 ± 1.8 g vs. 28.7 ± 3.9 g, *P* < 0.01). The body weight in the model group was lower compared to that in the model control group at the end of the study course (26.4 ± 2.7 g vs. 32.0 ± 6.1 g, *P* < 0.01). Atorvastatin, rapamycin, and GBE (200 mg/kg/day) did not affect the body weight of diabetic mice.

There was a significant increase in the plasma glucose level induced by STZ in the model group compared to the model control group (model group vs. model control group: 14.7 ± 2.1 mmolmmol/L vs. 7.4 ± 1.0 mmol/L, *P* < 0.01) and normal group (model group vs. normal group: 14.7 ± 2.1 mmol/L vs. 5.3 ± 0.8 mmol/L, *P* < 0.01) detected by a rapid blood glucose meter using the tail blood sample. After 12-week gastric administration, compared to the model group, GBE at both doses of 200 mg/kg/day and 400 mg/kg/day decreased the plasma glucose level [(200 mg/kg/day GBE group vs. model group: 18.8 ± 6.5 mmol/L vs. 23.4 ± 6.4 mmol/L, *P* < 0.01); (400 mg/kg/day GBE group vs. model group: 15.3 ± 7.1 mmol/l vs. 23.4 ± 6.4 mmol/l, *P* < 0.05)].

### 3.2. The Effects of GBE on Serum Lipid and Glucose Profiles

**Figure 5:**
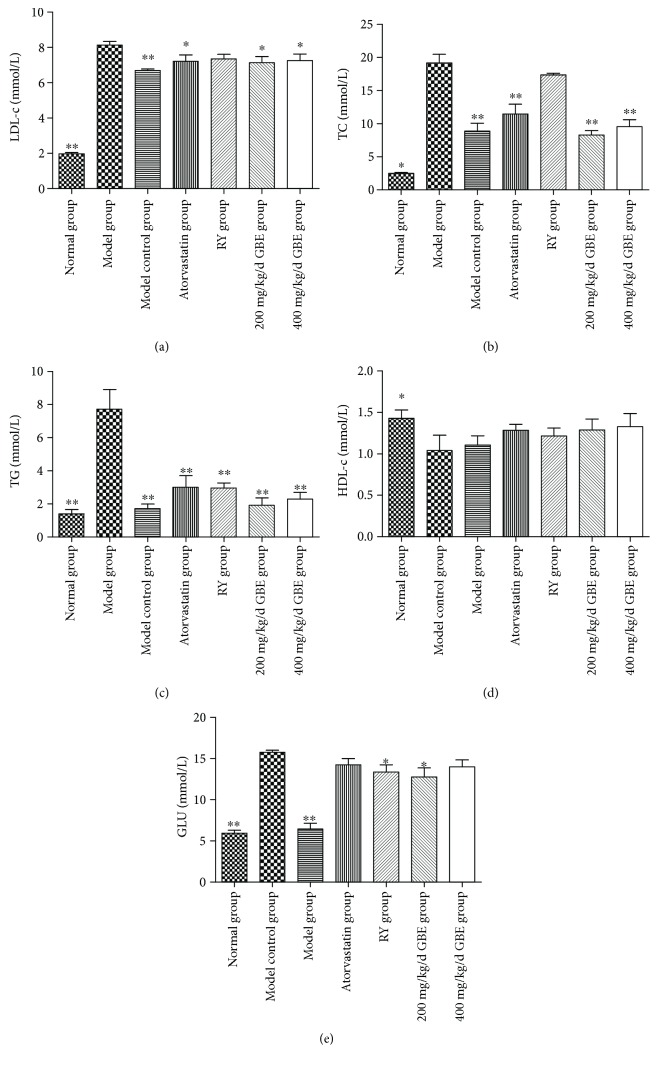
Effects of GBE on serum lipid and glucose profiles. ^∗^*P* < 0.05 and ^∗∗^*P* < 0.01, compared to the model group. GBE: ginkgo biloba leaf extract (normal group *n* = 18, model group *n* = 7, model control group *n* = 7, atorvastatin group *n* = 11, rapamycin group *n* = 15, 200 mg/kg/day GBE group *n* = 10, and 400 mg/kg/day GBE group *n* = 12).

The serum LDL-c, TC, TG, and glucose levels were significantly elevated in the model group compared to the normal group and model control group (*P* < 0.05, Figures 5(a)–5(c)), whereas the HDL-c level in the model group was lower in the model group compared to the normal group (*P* < 0.05, Figure 5(d)). Compared to the model group, atorvastatin and GBE (200 mg/kg/day and 400 mg/kg/day) significantly reduced the levels of LDL-c, TC, and TG (*P* < 0.05, Figures 5(a)–5(c)), without affecting the HDL-c level. Compared to the model group, rapamycin did not affect the serum levels of LDL-c, TC, and HDL-c (*P* > 0.05, Figures 5(a), 5(b), and 5(d)), whereas rapamycin decreased the serum TG level (rapamycin group vs. model group: 7.7 ± 3.2 mmol/L vs. 3.0 ± 1.2 mmol/L, *P* < 0.05) (Figure 5(c)). The serum TG level in the rapamycin group was higher than that in the model control group, but the difference was not statistically significant (*P* > 0.05). The serum glucose level was higher in the model group compared to the normal group and model control group; rapamycin and 200 mg/kg/day GBE decreased the serum glucose level of diabetic mice (*P* < 0.05, Figure 5(e)), but the levels were still higher than those in the model control group (*P* < 0.05).

### 3.3. The Effects of GBE on Serum Inflammatory Cytokines

**Figure 6:**
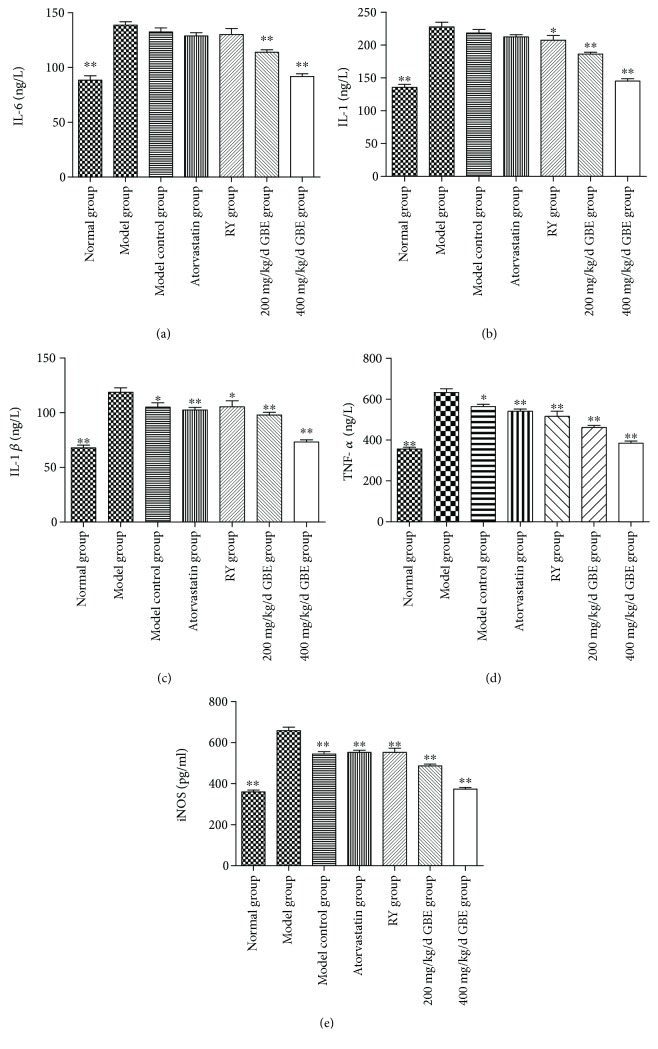
Effects of GBE on serum inflammatory cytokines. The level of IL-6, IL-1, IL-1*β*, TNF-*α*, and iNOS were determined by ELISA. ^∗^*P* < 0.05 and ^∗∗^*P* < 0.01, compared to the model group (normal group *n* = 18; model group *n* = 7, model control group *n* = 7, atorvastatin group *n* = 12, rapamycin group *n* = 15, 200 mg/kg/day GBE group *n* = 10, and 400 mg/kg/day GBE group *n* = 12).

The serum inflammatory cytokines including IL-1*β*, TNF-*α*, and iNOS were significantly elevated in the model group compared to the normal group and model control group (*P* < 0.05, Figures 5(c)–5(e)). The levels of IL-1 and IL-6 in the model group and model control group were significantly higher than that in the normal group (*P* < 0.05); the difference between the model group and the model control group was not statistically significant (*P* > 0.05, Figures 6(a) and 6(b)). GBE at both doses of 200 mg/kg/day and 400 mg/kg/day reduced the serum levels of IL-6, IL-1, IL-1*β*, TNF-*α*, and iNOS of diabetic mice (*P* < 0.01, Figures 6(a)–6(e)). Atorvastatin and rapamycin significantly decreased the serum levels of IL-1*β*, TNF-*α*, and iNOS (*P* < 0.01, Figures 6(c)–6(e)). Furthermore, rapamycin has been revealed to decrease the serum levels of IL-1 (*P* < 0.05, Figure 5(b)).

### 3.4. The Effects of GBE on Plaque Lipid Disposition

**Figure 7:**
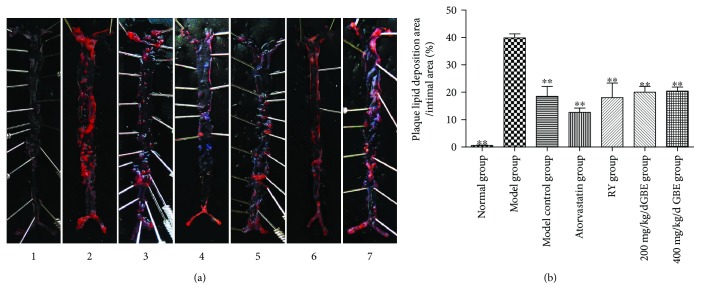
Effects of GBE on plaque lipid disposition. (a) Red “O” staining for en face analysis. (b) Plaque lipid disposition area/intima area. ^∗∗^*P* < 0.01, compared to the model group. (1) Normal group (*n* = 3), (2) model group (*n* = 3), (3) model control group (*n* = 3), (4) atorvastatin group (*n* = 3), (5) rapamycin group (*n* = 3), (6) 200 mg/kg/day GBE group (*n* = 3), and (7) 400 mg/kg/day GBE group (*n* = 3).

Compared to the model control group, plaque lipid disposition area/intima area in the model group was significantly increased; atorvastatin, rapamycin, and GBE (200 mg/kg/day and 400 mg/kg/day) decreased the plaque lipid disposition area/intima area, and the difference was statistically different (*P* < 0.05) (Figures 7(a) and 7(b)). The effect of 200 mg/kg/day and 400 mg/kg/day GBE on the plaque lipid disposition area/intima area was not significantly different.

### 3.5. The Effect of GBE on Plaque Area and Collagen Area

**Figure 8:**
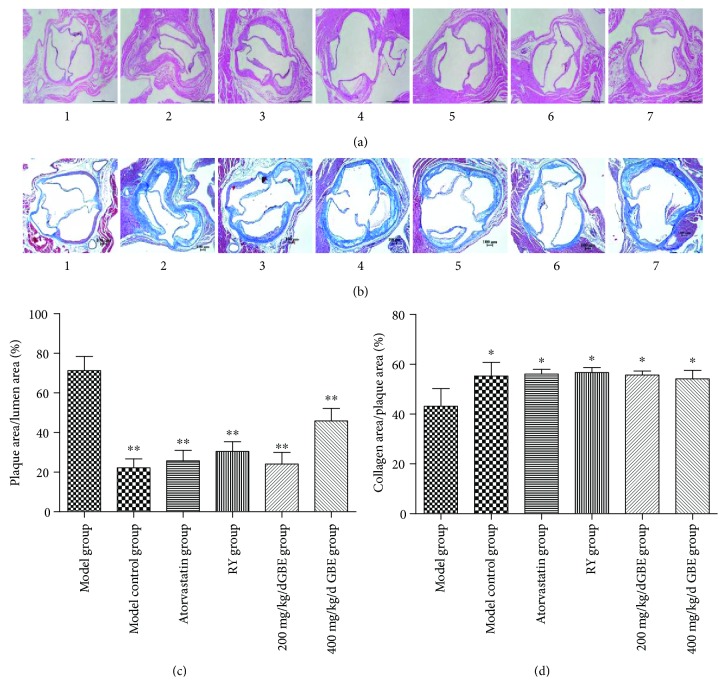
Effects of GBE on plaque area/lumen area and collagen area/plaque area. (a, c) HE staining for determination of plaque area/lumen area; (b, d) MASSON staining for determination of collagen area/plaque area. ^∗^*P* < 0.05 and ^∗∗^*P* < 0.01, compared to the model group. (1) Normal group (*n* = 5), (2) model group (*n* = 3), (3) model control group (*n* = 3), (4) atorvastatin group (*n* = 4), (5) rapamycin group (*n* = 5), (6) 200 mg/kg/day GBE group (*n* = 4), and (7) 400 mg/kg/day GBE group (*n* = 4). (a, b) 40x.

Compared to the model control group, the plaque area/lumen area in the model group was significantly increased in the model group, whereas the collagen area/plaque area in the model group was significantly reduced. Atorvastatin, rapamycin, and GBE (200 mg/kg/day and 400 mg/kg/day) significantly (200 mg/kg/day and 400 mg/kg/day) decreased the plaque area/lumen area (*P* < 0.05, Figures 8(a) and 8(c)). On the contrary, they increased the collagen area/plaque area (*P* < 0.05, Figures 8(b) and 8(d)). Compared to the model control group, the effects of GBE at 200 mg/kg/day or 400 mg/kg/day on the plaque area/lumen area and collagen area/plaque area were not statistically different.

### 3.6. The Effect of GBE on Inflammatory Markers and *α*-SMA in the Atherosclerotic Plaques

**Figure 9:**
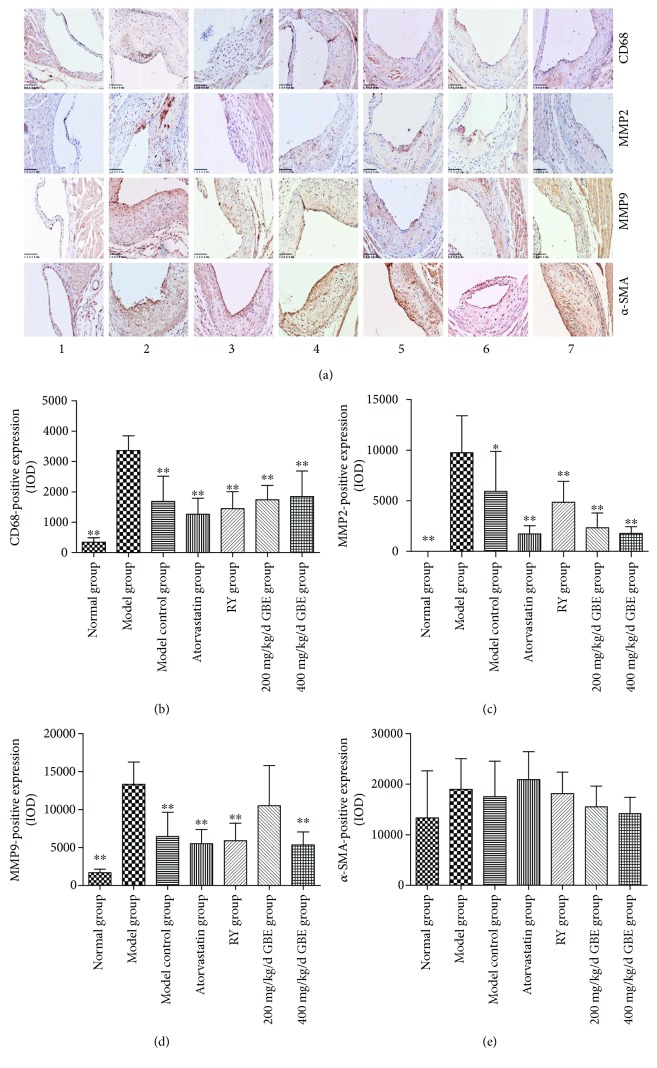
Immunohistochemical staining for the expression of CD68, MMP2, MMP9 and *α*-SMA in the atherosclerotic plaques. ^∗∗^*P* < 0.05, ^∗∗^*P* < 0.01, compared to model group. (1) Normal group (*n* = 3), (2) model group (*n* = 5), (3) model control group (*n* = 5), (4) atorvastatin group (*n* = 5), (5) rapamycin group (*n* = 5), (6) 200 mg/kg/day GBE group (*n* = 5), and (7) 400 mg/kg/day GBE group (*n* = 5) (immunohistochemical staining 200x).

Compared to the normal group and model control group, the expressions of CD68, MMP2, and MMP9 were significantly increased in the model group; atorvastatin, rapamycin, and GBE (200 mg/kg/day and 400 mg/kg/day) significantly decreased the expression of CD68, MMP2, and MMP9 in the plaques (*P* < 0.05, Figures 9(a)–9(d)). There was no statistical difference among the groups according to the expression of *α*-SMA. There was an increasing trend in the expression of *α*-SMA when the diabetic mice were treated with atorvastatin (*P* = 0.074, Figure 9(e)).

### 3.7. GBE Attenuates Endoplasmic Reticulum Stress by Upregulating Autophagy

**Figure 10:**
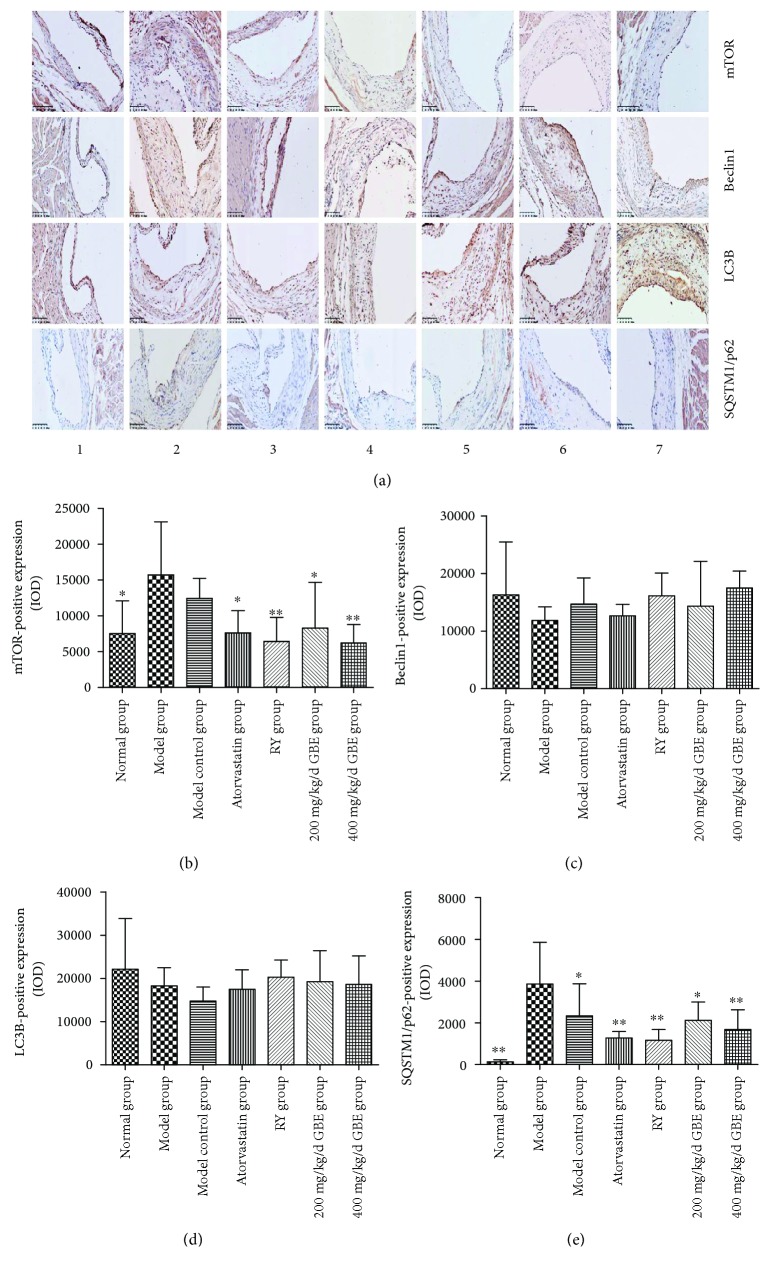
Effects of GBE on autophagy hallmark expressions in the atherosclerotic plaques. Immunohistochemical staining for the expression of mTOR, Beclin1, LC3B, and SQSTM1/p62 in the plaques. ^∗^*P* < 0.05 and ^∗∗^*P* < 0.01, compared to the model group. (1) Normal group (*n* = 3), (2) Model group (*n* = 5), (3) model control group (*n* = 5), (4) atorvastatin group (*n* = 5), (5) rapamycin group (*n* = 5), (6) 200 mg/kg/day GBE group (*n* = 5), and (7) 400 mg/kg/day GBE group (*n* = 5) (immunohistochemical staining 200x).

**Figure 11:**
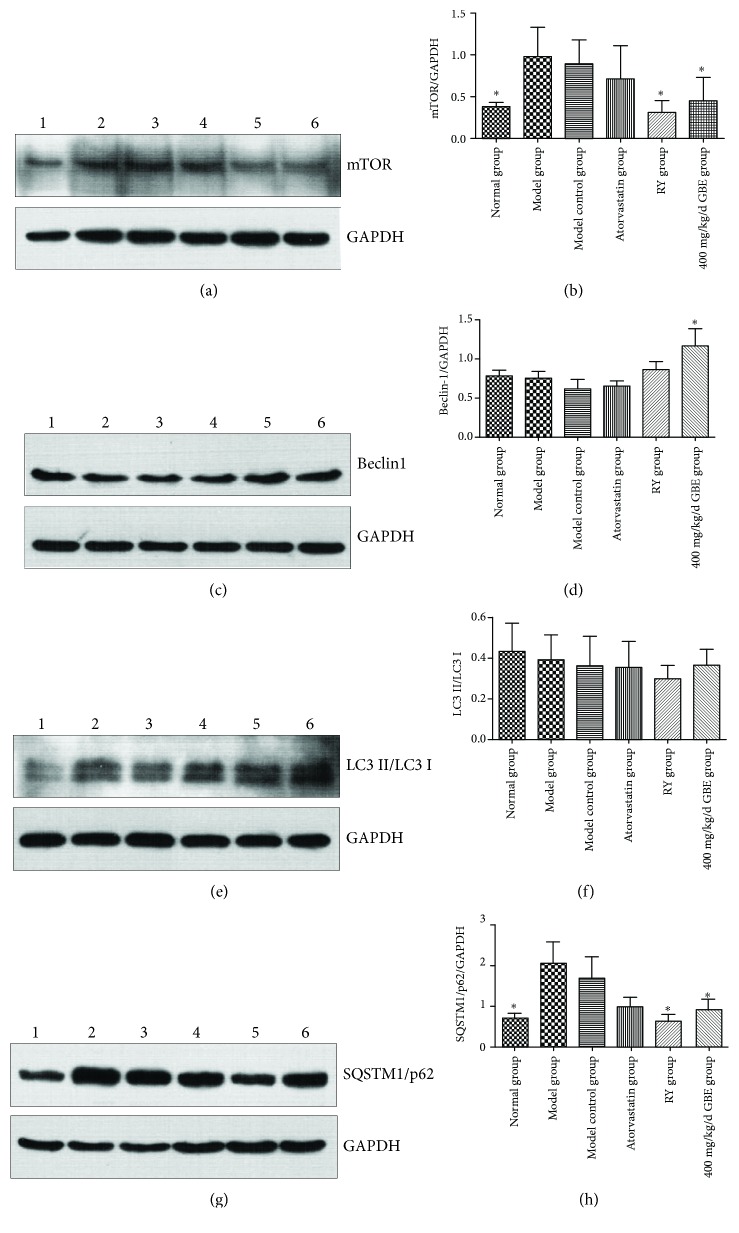
Effects of GBE on autophagy hallmark expressions in the aorta of mice. The expression of mTOR, Beclin1, LC3II/LC3I, and SQSTM1/p62 in the aorta was determined by Western blotting. (a, b) The expression of mTOR (*n* = 3 in each group); (c, d) the expression of Beclin1 (*n* = 6 in each group); (e, f) the expression of LC3II/LC3I (*n* = 5 in each group); (g, h) the expression of SQSTM1/p62 (*n* = 3 in each group). ^∗^*P* < 0.05, compared to the model group. (1) Normal group, (2) model group, (3) model control group, (4) atorvastatin group, (5) rapamycin group, (6) 200 mg/kg/day GBE group, and (7) 400 mg/kg/day GBE group.

Immunohistochemical staining and Western blotting revealed that the expression of mTOR was significantly increased in the model group and model control group compared to the normal group (*P* < 0.05, Figures 10(a), 10(b), 11(a), and 11(b)). Rapamycin, and GBE (200 mg/kg/day and 400 mg/kg/day) significantly inhibited the increased expression of mTOR in diabetic mice (*P* < 0.05, Figures 10(a), 10(b), 11(a), and 11(b)). There was an increasing trend in the expressions of mTOR in atherosclerotic plaques and aorta of the model group compared to the model control group, but the difference was not statistically significant (*P* > 0.05, Figures 10(a), 10(b), 11(a), and 11(b)).

There were no significant differences among the groups according to the expressions of LC3B and Beclin1 in the atherosclerotic plaques analyzed by immunohistochemical staining (Figures 10(a), 10(c), and 10(d)). There was an increasing trend in Beclin1 expression in the atherosclerotic plaques of the GBE group compared to the model group (*P* = 0.081, Figures 10(a) and 10(c)). Western blotting showed that there was no statistically significant difference between the model group and model control group according to the expressions of Beclin1 and LC3II/LC3I in the aortas (*P* > 0.05, Figures 11(c)–11(f)). Western blotting showed that GBE at a dose of 400 mg/kg/day significantly increased the expression of Beclin1 in aortas compared to the model group (*P* < 0.05, Figures 11(c) and 11(d)).

Immunohistochemical staining showed that the expression of SQSTM1/p62 in the atherosclerotic plaques of the model group significantly increased compared to the model control group (*P* < 0.05, Figures 10(a) and 10(e)). Western blotting displayed that there was a high level of SQSTM1/p62 in the aorta of the model group compared to the normal group and model control group (*P* < 0.05, Figures 11(g) and 11(h)). There was an increasing trend in the expression of SQSTM1/p62 in the aorta of the model group when compared to the model control group, but the difference was not statistically different. Rapamycin and GBE (400 mg/kg/day) inhibited the SQSTM1/p62 expression in atherosclerotic plaques and aortas of diabetic mice (*P* < 0.05, Figures 10(a), 10(e), 11(g), and 11(h)). Atorvastatin has a trend to decrease the expression of SQSTM1/p62 in the aorta, but the difference was not statistically different (Figures 11(g) and 11(h), *P* = 0.061).

**Figure 12:**
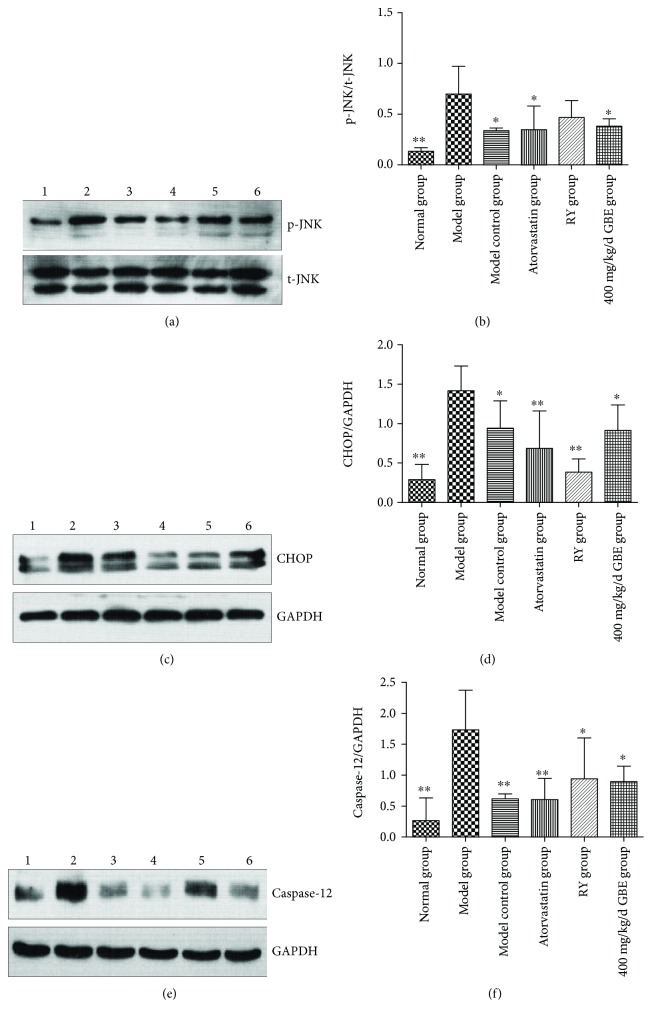
Effects of GBE on endoplasmic reticulum stress hallmark expressions in the aortas of mice. The expressions of p-JNK, CHOP, and Caspase-12 were determined by Western blotting. (a, b) The expression of p-JNK (*n* = 3 in each group); (c, d) the expression of CHOP (*n* = 4 in each group); (e, f) the expression of Caspase-12 (*n* = 3 in each group). ^∗^*P* < 0.05 and ^∗∗^*P* < 0.01, compared to the model group. (1) Normal group, (2) model group, (3) model control group, (4) atorvastatin group, (5) rapamycin group, (6) 200 mg/kg/day GBE group, and (7) 400 mg/kg/day GBE group.

Western blot revealed that the expressions of endoplasmic reticulum stress markers including p-JNK, CHOP, and Caspase-12 in the aortas of the model group were higher than those in the model control group (*P* < 0.05, Figures 12(a)–12(f)); atorvastatin and GBE at a dose of 400 mg/kg/day decreased the expressions of p-JNK, CHOP, and Caspase-12 (*P* < 0.05, Figures 12(a)–12(f)). Rapamycin decreased the expression of CHOP and Caspase-12 (*P* < 0.05, Figures 12(c)–12(f)); the difference of the p-JNK level was not statistically different (*P* > 0.05, Figures 12(a) and 12(b)).

## 4. Discussion

Endoplasmic reticulum stress is a common pathological mechanism for diabetes mellitus and atherosclerosis; thus, attenuating endoplasmic reticulum stress is one of the important strategies for inhibition of diabetes mellitus and diabetic vascular complications. Endoplasmic reticulum stress has been exposed to upregulated autophagy, which in turn protectively inhibits unfolded protein response in a feedback manner [41]. Atherosclerosis is one of the main complications of diabetes mellitus. STZ-induced ApoE-/- mice were commonly used to establish diabetic atherosclerosis models [42]. Network pharmacology provides a new approach for exploring drug targets. The network pharmacology utilized in the present study predicted that ginkgo biloba leaf attenuates atherosclerosis through mTOR and NF-*κ*B-mediated inflammation signaling pathways. Animal experiments showed that high glucose accelerated atherosclerotic plaques, characterized by an increase in both plaque area/lumen area and plaque lipid disposition area/intima area and a decrease in the collagen area/plaque area. Furthermore, the increased expressions of CD68, MMP2, and MMP9 in the atherosclerotic plaques were induced by high glucose. High glucose promotes the expression of endoplasmic reticulum stress markers including p-JNK, CHOP, and Caspase-12. The expressions of mTOR and SQSTM1/p62 were upregulated in the aorta of the model group compared to the normal group. In addition, the expression of SQSTM1/p62 in the atherosclerotic plaques of the model group was higher than that in the model control group. mTOR inhibitor rapamycin and GBE stabilized atherosclerotic plaques, featured by a decreased plaque area/lumen area and plaque lipid disposition area/intima area. The expressions of CD68, MMP2, and MMP9 in the atherosclerotic plaques were decreased by rapamycin and GBE. Rapamycin and GBE inhibited the SQSTM1/p62 expression of diabetic ApoE-/- mice and, consequently, reduced the expression of endoplasmic reticulum stress markers, including p-JNK, CHOP, and Caspase-12, via inhibition of the mTOR-dependent signaling pathway. GBE reduced serum glucose and inflammatory cytokines including IL-1, IL-6, IL-1*β*, TNF-*α*, and iNOS. Consistent with the previous studies, the TC, LD, and TG levels were increased in STZ-induced ApoE-/mice compared to ApoE-/- mice and C57BL/6J mice [42]. GBE has a positive regulating effect on the lipid profiles.

Moderate autophagy has the ability to prevent the onset and progression of diabetes mellitus and atherosclerosis [43, 44]. Overactivation of mTOR is linked to impaired autophagy. It has been demonstrated that metformin, an agent used to control hyperglycemia in diabetes mellitus, inhibits mTOR activity and promotes autophagy and protects against endothelial cell senescence [45, 46]. The present study showed that mTOR is activated in the aorta of diabetic ApoE-/- mice compared to C57BL/6J mice; furthermore, there was an increasing trend in the expression of mTOR in the atherosclerotic plaques and aortas of the model group compared to the model control group. As mentioned above, SQSTM1/p62 accumulation is closely associated with impaired autophagy. The present study revealed that the expression of SQSTM1/p62 in the aortas of diabetic ApoE-/- mice and nondiabetic ApoE-/- mice were both increased compared to C57BL/6J mice; additionally, SQSTM1/p62 was upregulated in atherosclerotic plaques of diabetic ApoE-/- mice compared to nondiabetic ApoE-/- mice. These findings were supported by the study of Fetterman et al. [25] that inadequate autophagy contributes to endothelial dysfunction in patients with diabetes. According to them, SQSTM1/p62 aggregated in endothelia indicating an impaired autophagy; LC3II has shown an increasing trend in a feedback manner. We also showed that rapamycin and GBE inhibited the expression of SQSTM1/p62 which increased in diabetic mice via blocking of mTOR signaling. Collectively, overactive mTOR and SQSTM1/p62 contribute to diabetic atherosclerosis [12, 25, 27, 28]. The downregulating effect of rapamycin on SQSTM1/p62 was coincident with the previous study [28] that high glucose promotes the activation of mTOR signaling, while rapamycin inhibited the accumulation of SQSTM1/p62 and microvascular endothelial cell apoptosis induced by high glucose by blockage of the upregulation of mTOR. Furthermore, the effect of GBE on autophagy in the present study supported by Zhang et al. [47] that luteolin, one of the active compounds of GBE which attenuate foam cell formation and macrophage apoptosis by promoting autophagy.

**Figure 13:**
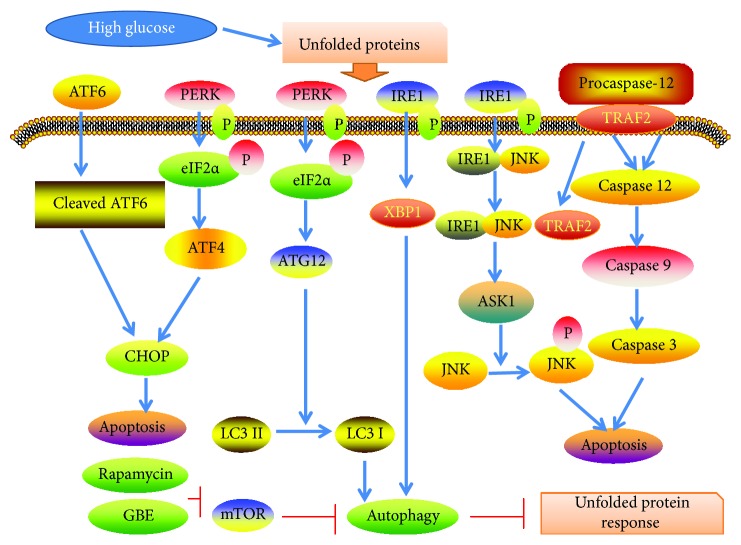
The mechanism by which GBE attenuates diabetic atherosclerosis. GBE attenuates unfolded protein response by upregulation of autophagy via inhibition of mTOR.

Endoplasmic reticulum stress-mediated apoptosis accelerates diabetic atherosclerosis. Autophagy is required to remove misfolded proteins and eliminate nonfunctioning mitochondria. Endoplasmic reticulum stress markers including p-JNK, CHOP, and Caspase-12 were increased in diabetic ApoE-/- mice in the present study, supported by previous studies [48, 49]. Rapamycin and GBE inhibited the expressions of p-JNK, CHOP, and Caspase-12 by reducing SQSTM1/p62 via inhibition of mTOR signaling, indicating a protective role of autophagy in diabetic atherosclerosis (Figure 13).

High glucose, representing a disordered metabolism, is also a chronic inflammatory process. Endoplasmic reticulum stress which was upregulated in the context of high glucose activates NF-*κ*B by its IRE1*α* and PERK signaling, attributable to macrophage adhesion to the vascular wall and development of atherosclerosis [50]. These inflammatory cytokines increased in the diabetic condition, promote the biogenesis of ROS, and activate NF-*κ*B in a positive feedback manner, leading to further generation of inflammatory cytokines [49, 50]. Atorvastatin, rapamycin, and GBE attenuated diabetes-induced endoplasmic reticulum stress in diabetic ApoE-/- mice, blocking the positive feedback loop of endoplasmic reticulum stress and inflammation, which consequently reduced the inflammation in vivo, characterized by decreased inflammation cytokines, and decreased infiltration of macrophages and inhibited MMP expression. The reduced expression of MMP by GBE is probably attributable for attenuation of endoplasmic reticulum stress or inhibition of SQSTM1/p62 [27].

The present study revealed that GBE downregulated the blood glucose level. According to previous studies, the mechanisms by which GBE reduced blood glucose involved antioxidant, pancreas protection, and insulin sensitivity enhancement [51, 52]. In the present study, 400 mg/kg/day GBE reduced the plasma glucose level, whereas it did not affect the serum glucose level, which might be attributable to different detecting methods and the predisposition to stress response in the model group mice when tail blood samples were obtained. The mechanisms that cause different effects of 400 mg/kg/day GBE on plasma glucose and serum glucose level need further investigation. Furthermore, the attenuation of endoplasmic reticulum stress in the present study was probably another mechanism for GBE against hyperglycemia. The findings about the effect of rapamycin on blood glucose are controversial. It has been shown that activation of mTOR contributes to the secretion of insulin and increase in insulin sensitivity. On the contrary, other studies revealed that enhancing macrophage autophagy by the mTOR inhibitor may be a viable therapeutic or preventative approach to inflammatory disease, obesity insulin resistance, and diabetes [44, 53]. Interestingly, the present study showed that rapamycin at a dose of 1 mg/kg/day exerts a positive effect on serum glucose in STZ plus high-fat diet-induced diabetic ApoE-/- mice, and the finding was supported by den Hartigh et al. [16]. Further studies needed to investigate the effect of rapamycin on blood glucose in STZ plus high-fat diet-induced ApoE-/- mice.

According to Elloso et al. [54], ApoE-/- mice dosed q.o.d. with 1, 2, 4, or 8 mg/kg of sirolimus for 13 weeks associated with approximately 30% increase in LDL-c, regardless of the dosage implemented. Sirolimus treatment of 8 mg/kg was associated with an increase in HDL-c of more than 40 mg/dL. Zhao et al. [38] showed that low-dose oral sirolimus (0.1 mg/kg, 0.3 mg/kg, and 1 mg/kg for 8 or 16 weeks) effectively delayed the progression of atherosclerosis and modulated the plaque phenotype in LDL-rKO mice, without influencing total lipid levels, indicating that low-dose oral sirolimus is well tolerated and exerts a potent antiatherosclerotic effect. The present study showed that low dose of rapamycin (1 mg/kg/day) did not affect LDL-c and TC levels of diabetic ApoE-/- mice; low dose of rapamycin reduced the TG level compared to the model group, but the level was still higher than that in the model control group (*P* > 0.05). Therefore, the downregulating effect of GBE on TG was probably attributable to glucose metabolism improvement. The lipid regulation effect of GBE against hyperlipidemia was consistent with the study of Wei et al. [55] that GBE inhibited high-fat diet-induced increased of serum TG, TC, and LDL-c levels. According to Zhang et al. [56], GBE exerts multidirectional lipid-lowering effects by limitation of the absorption of cholesterol, inactivation of HMGCoA, and favorable regulation of profiles of essential polyunsaturated fatty acid.

## 5. Study Limitations

Firstly, the present animal experiment was not capable of verifying each active compound that protects against atherosclerosis included in network pharmacology. However, network pharmacology indeed provides a feasible approach for predicting the potential active compound and target. Due to the limited available information of ginkgolide, the antiatherosclerotic effect needs further investigation. The expressions of mTOR and SQSTM1/p62 were elevated in the aorta of both the model group and the model control group when compared to the normal group. Secondly, in the present study, beside a significantly increased expression of SQSTM1/p62 in the atherosclerotic plaques in the model group compared to the model group, there was an increasing trend of mTOR and SQSTM1/p62 expressions in the aorta of the model group compared to the model group, maybe due to a relatively small sample. However, we concluded that overactive mTOR and SQSTM1/p62 were contributors to atherosclerotic plaques. Thirdly, the dose-dependent manner of GBE against diabetic atherosclerosis was not found, and it was not clear whether there exists a dose-dependent manner in attenuation of diabetic atherosclerosis with the dose of GBE less than 200 mg/kg/day. Fourth, GBE at doses of 200-400 mg/kg/day may reach a platform in attenuation of diabetic atherosclerosis. The optimal dose of GBE for attenuation of diabetic atherosclerosis needs further investigation, because there was lack of study on the protective effect of diabetic atherosclerosis in vivo. Furthermore, GBE may reduce SQSTM1/p62 accumulation by activating the high glucose-inhibited AMPK upstream of mTOR. However, it did not affect the findings of the present study. The protective effect of mTOR inhibitor rapamycin against diabetic atherosclerosis indicated that GBE attenuated diabetic atherosclerosis and endoplasmic reticulum stress-mediated apoptosis by blockage of SQSTM1/p62 via direct inhibition of mTOR. Lastly, given the critical role of macrophage autophagy in atherosclerotic progression, further study should focus on the effect of GBE on autophagy and endoplasmic reticulum stress in macrophages.

## 6. Conclusions

Our study demonstrates that impaired autophagy was associated with diabetic atherosclerosis, characterized by upregulated mTOR expression and SQSTM1/p62 accumulation. Both mTOR inhibitor rapamycin and GBE protected against diabetic atherosclerosis by attenuation of endoplasmic reticulum through downregulation of SQSTM1/p62 by inhibiting mTOR signaling.

## Data Availability

All data generated or analyzed during this study are included in this published article.

## Abbreviations

GBE: Ginkgo biloba leaf extract
mTOR: Mammalian target of rapamycin
AGE: Advanced glycation end products
PI3K: Phosphatidylinositol 3 kinase
AMPK: AMP-activated protein kinase
IRS1: Insulin receptor substrate 1
CMECs: Cardiac microvascular endothelial cells
STZ: Streptozotocin.

## References

[1] Wang S., Wang Z., Fan Q. (2016). Ginkgolide K protects the heart against endoplasmic reticulum stress injury by activating the inositol-requiring enzyme 1*α*/X box-binding protein-1 pathway. *British Journal of Pharmacology*.

[2] Liao X., Sluimer J. C., Wang Y. (2012). Macrophage autophagy plays a protective role in advanced atherosclerosis. *Cell Metabolism*.

[3] Sun W., Lin Y., Chen L. (2018). Legumain suppresses OxLDL-induced macrophage apoptosis through enhancement of the autophagy pathway. *Gene*.

[4] Geng Z., Xu F., Zhang Y. (2016). miR-129-5p-mediated Beclin-1 suppression inhibits endothelial cell autophagy in atherosclerosis. *American Journal of Translational Research*.

[5] Ma J., Yang S., Ma A. (2017). Expression of miRNA-155 in carotid atherosclerotic plaques of apolipoprotein E knockout (ApoE^−/−^) mice and the interventional effect of rapamycin. *International Immunopharmacology*.

[6] Pakala R., Stabile E., Jang G. J., Clavijo L., Waksman R. (2005). Rapamycin attenuates atherosclerotic plaque progression in apolipoprotein E knockout mice: inhibitory effect on monocyte chemotaxis. *Journal of Cardiovascular Pharmacology*.

[7] Gadioli A. L. N., Nogueira B. V., Arruda R. M. P. (2009). Oral rapamycin attenuates atherosclerosis without affecting the arterial responsiveness of resistance vessels in apolipoprotein E-deficient mice. *Brazilian Journal of Medical and Biological Research*.

[8] Chiang C. K., Wang C. C., Lu T. F. (2016). Involvement of endoplasmic reticulum stress, autophagy, and apoptosis in advanced glycation end products-induced glomerular mesangial cell injury. *Scientific Reports*.

[9] Xie Y., You S. J., Zhang Y. L. (2011). Protective role of autophagy in AGE-induced early injury of human vascular endothelial cells. *Molecular Medicine Reports*.

[10] He C., Zhu H., Zhang W. (2013). 7-Ketocholesterol induces autophagy in vascular smooth muscle cells through Nox4 and Atg4B. *The American Journal of Pathology*.

[11] Harrington L. S., Findlay G. M., Gray A. (2004). The TSC1-2 tumor suppressor controls insulin-PI3K signaling via regulation of IRS proteins. *The Journal of Cell Biology*.

[12] Chong Z. Z., Maiese K. (2012). Mammalian target of rapamycin signaling in diabetic cardiovascular disease. *Cardiovascular Diabetology*.

[13] Khamzina L., Veilleux A., Bergeron S., Marette A. (2005). Increased activation of the mammalian target of rapamycin pathway in liver and skeletal muscle of obese rats: possible involvement in obesity-linked insulin resistance. *Endocrinology*.

[14] Ebato C., Uchida T., Arakawa M. (2008). Autophagy is important in islet homeostasis and compensatory increase of beta cell mass in response to high-fat diet. *Cell Metabolism*.

[15] Su J., Zhou L., Kong X. (2013). Endoplasmic reticulum is at the crossroads of autophagy, inflammation, and apoptosis signaling pathways and participates in the pathogenesis of diabetes mellitus. *Journal Diabetes Research*.

[16] den Hartigh L. J., Goodspeed L., Wang S. A. (2018). Chronic oral rapamycin decreases adiposity, hepatic triglycerides and insulin resistance in male mice fed a diet high in sucrose and saturated fat. *Experimental Physiology*.

[17] Jessen N., Koh H. J., Folmes C. D. (2010). Ablation of LKB1 in the heart leads to energy deprivation and impaired cardiac function. *Biochimica et Biophysica Acta*.

[18] Zang M., Xu S., Maitland-Toolan K. A. (2006). Polyphenols stimulate AMP-activated protein kinase, lower lipids, and inhibit accelerated atherosclerosis in diabetic LDL receptor-deficient mice. *Diabetes*.

[19] Yang J., Wang N., Zhu Y., Feng P. (2011). Roles of SIRT1 in high glucose-induced endothelial impairment: association with diabetic atherosclerosis. *Archives of Medical Research*.

[20] Jin X., Chen M., Yi L. (2014). Delphinidin-3-glucoside protects human umbilical vein endothelial cells against oxidized low-density lipoprotein-induced injury by autophagy upregulation via the AMPK/SIRT1 signaling pathway. *Molecular Nutrition & Food Research*.

[21] Yang X., Wei J., He Y. (2017). SIRT1 inhibition promotes atherosclerosis through impaired autophagy. *Oncotarget*.

[22] Guo W., Qian L., Zhang J. (2011). Sirt1 overexpression in neurons promotes neurite outgrowth and cell survival through inhibition of the mTOR signaling. *Journal of Neuroscience Research*.

[23] Zhang S., Cai G., Fu B. (2012). SIRT1 is required for the effects of rapamycin on high glucose-inducing mesangial cells senescence. *Mechanisms of Ageing and Development*.

[24] Hou J., Zheng D., Xiao W., Li D., Ma J., Hu Y. (2018). Mangiferin enhanced autophagy via inhibiting mTORC1 pathway to prevent high glucose-induced cardiomyocyte injury. *Frontiers in Pharmacology*.

[25] Fetterman J. L., Holbrook M., Flint N. (2016). Restoration of autophagy in endothelial cells from patients with diabetes mellitus improves nitric oxide signaling. *Atherosclerosis*.

[26] Razani B., Feng C., Coleman T. (2012). Autophagy links inflammasomes to atherosclerotic progression. *Cell Metabolism*.

[27] Zhou F., Liu D., Ning H. F., Yu X. C., Guan X. R. (2016). The roles of p62/SQSTM1 on regulation of matrix metalloproteinase-9 gene expression in response to oxLDL in atherosclerosis. *Biochemical and Biophysical Research Communications*.

[28] Zhang Z., Zhang S., Wang Y. (2017). Autophagy inhibits high glucose induced cardiac microvascular endothelial cells apoptosis by mTOR signal pathway. *Apoptosis*.

[29] Wang D., Yu W., Liu Y. (2017). Roles of autophagy in ischemic heart diseases and the modulatory effects of Chinese herbal medicine. *The American Journal of Chinese Medicine*.

[30] van Beek T. A. (2002). Chemical analysis of Ginkgo biloba leaves and extracts. *Journal of Chromatography. A*.

[31] Tosaki A., Pali T., Droy-Lefaix M. T. (1996). Effects of Ginkgo biloba extract and preconditioning on the diabetic rat myocardium. *Diabetologia*.

[32] Tian J., Liu Y., Liu Y., Chen K., Lyu S. (2018). *Ginkgo biloba* leaf extract protects against myocardial injury via attenuation of endoplasmic reticulum stress in streptozotocin-induced diabetic ApoE^−/−^ mice. *Oxidative Medicine and Cellular Longevity*.

[33] Chen J. S., Chen Y. H., Huang P. H. (2012). Ginkgo biloba extract reduces high-glucose-induced endothelial adhesion by inhibiting the redox-dependent interleukin-6 pathways. *Cardiovascular Diabetology*.

[34] Huang J., Cheung F., Tan H. Y. (2017). Identification of the active compounds and significant pathways of yinchenhao decoction based on network pharmacology. *Molecular Medicine Reports*.

[35] Geoffrion M., Du X., Irshad Z. (2014). Differential effects of glyoxalase 1 overexpression on diabetic atherosclerosis and renal dysfunction in streptozotocin-treated, apolipoprotein E-deficient mice. *Physiological Reports*.

[36] Pan Y., Wang Y., Zhao Y. (2014). Inhibition of JNK phosphorylation by a novel curcumin analog prevents high glucose-induced inflammation and apoptosis in cardiomyocytes and the development of diabetic cardiomyopathy. *Diabetes*.

[37] Nie P., Li D., Hu L. (2014). Atorvastatin improves plaque stability in ApoE-knockout mice by regulating chemokines and chemokine receptors. *PLoS One*.

[38] Zhao L., Ding T., Cyrus T. (2009). Low-dose oral sirolimus reduces atherogenesis, vascular inflammation and modulates plaque composition in mice lacking the LDL receptor. *British Journal of Pharmacology*.

[39] Liu Y., Liu Y. F., Tian J. F., Fu C. G., Chen K. J. (2017). The effect and mechanism of extract of Ginkgo biloba (EGb) on cardiovascular protection on the rat model of type 2 diabetes after myocardial infarction. *Traditional and Western Medicine*.

[40] Qiao Z. Y., Huang J. H., Ma J. W. (2014). Ginkgo biloba extract reducing myocardium cells apoptosis by regulating apoptotic related proteins expression in myocardium tissues. *Molecular Biology Reports*.

[41] Tang Q., Zheng G., Feng Z. (2017). Trehalose ameliorates oxidative stress-mediated mitochondrial dysfunction and ER stress via selective autophagy stimulation and autophagic flux restoration in osteoarthritis development. *Cell Death & Disease*.

[42] Wu K. K., Huan Y. (2007). Diabetic atherosclerosis mouse models. *Atherosclerosis*.

[43] Luo Z., Xu W., Ma S. (2017). Moderate autophagy inhibits vascular smooth muscle cell senescence to stabilize progressed atherosclerotic plaque via the mTORC1/ULK1/ATG13 signal pathway. *Oxidative Medicine and Cellular Longevity*.

[44] Kang Y. H., Cho M. H., Kim J. Y. (2016). Impaired macrophage autophagy induces systemic insulin resistance in obesity. *Oncotarget*.

[45] Arunachalam G., Samuel S. M., Marei I., Ding H., Triggle C. R. (2014). Metformin modulates hyperglycaemia-induced endothelial senescence and apoptosis through SIRT1. *British Journal of Pharmacology*.

[46] Maiese K. (2015). mTOR: driving apoptosis and autophagy for neurocardiac complications of diabetes mellitus. *World Journal of Diabetes*.

[47] Zhang B. C., Zhang C. W., Wang C., Pan D. F., Xu T. D., Li D. Y. (2016). Luteolin attenuates foam cell formation and apoptosis in ox-LDL-stimulated macrophages by enhancing autophagy. *Cellular Physiology and Biochemistry*.

[48] Lin Y., Zhuang J., Li H. (2016). Vaspin attenuates the progression of atherosclerosis by inhibiting ER stress-induced macrophage apoptosis in apoE/mice. *Molecular Medicine Reports*.

[49] Dong Y., Fernandes C., Liu Y. (2017). Role of endoplasmic reticulum stress signalling in diabetic endothelial dysfunction and atherosclerosis. *Diabetes and Vascular Disease Research*.

[50] Ramji D. P., Davies T. S. (2015). Cytokines in atherosclerosis: key players in all stages of disease and promising therapeutic targets. *Cytokine & Growth Factor Reviews*.

[51] Cheng D., Liang B., Li Y. (2013). Antihyperglycemic effect of *Ginkgo biloba* extract in streptozotocin-induced diabetes in rats. *BioMed Research International*.

[52] Hirata B. K. S., Banin R. M., Dornellas A. P. S. (2015). *Ginkgo biloba* extract improves insulin signaling and attenuates inflammation in retroperitoneal adipose tissue depot of obese rats. *Mediators of Inflammation*.

[53] Liu K., Zhao E., Ilyas G. (2015). Impaired macrophage autophagy increases the immune response in obese mice by promoting proinflammatory macrophage polarization. *Autophagy*.

[54] Elloso M. M., Azrolan N., Sehgal S. N. (2003). Protective effect of the immunosuppressant sirolimus against aortic atherosclerosis in apo E-deficient mice. *American Journal of Transplantation*.

[55] Wei J. M., Wang X., Gong H., Shi Y. J., Zou Y. (2013). Ginkgo suppresses atherosclerosis through downregulating the expression of connexin 43 in rabbits. *Archives of Medical Science*.

[56] Zhang Q., Wang G. J., A J. Y. (2009). Application of GC/MS-based metabonomic profiling in studying the lipid-regulating effects of *Ginkgo biloba* extract on diet-induced hyperlipidemia in rats. *Acta Pharmacologica Sinica*.

