# Erratum to “Ginkgo Biloba Leaf Extract Attenuates Atherosclerosis in Streptozotocin-Induced Diabetic ApoE-/- Mice by Inhibiting Endoplasmic Reticulum Stress via Restoration of Autophagy through the mTOR Signaling Pathway”

**DOI:** 10.1155/2019/3084083

**Published:** 2019-09-10

**Authors:** Jinfan Tian, Mohammad Sharif Popal, Yanfei Liu, Rui Gao, Shuzheng Lyu, Keji Chen, Yue Liu

**Affiliations:** ^1^Department of Cardiology, Beijing Anzhen Hospital, Capital Medical University, Beijing Institute of Heart, Lung and Blood Vessel Diseases, Beijing 100029, China; ^2^Cardiovascular Disease Center, Xiyuan Hospital, China Academy of Chinese Medical Sciences, Beijing 100091, China; ^3^Graduate School, Beijing University of Chinese Medicine, Beijing 100029, China; ^4^Institute of Clinical Pharmacology of Xiyuan Hospital, China Academy of Chinese Medical Sciences, Beijing 100091, China

In the article titled “Ginkgo Biloba Leaf Extract Attenuates Atherosclerosis in Streptozotocin-Induced Diabetic ApoE-/- Mice by Inhibiting Endoplasmic Reticulum Stress via Restoration of Autophagy through the mTOR Signaling Pathway” [1], there was an error in the group labels of Figures 5(d) and 5(e), where the second and third group labels in each image were reversed. Additionally, there was an error in Figure 5(b) where the symbol “∗” should be corrected to “∗∗” in the Normal group bar. The corrected figure is shown below.

## Figures and Tables

**Figure 1:**
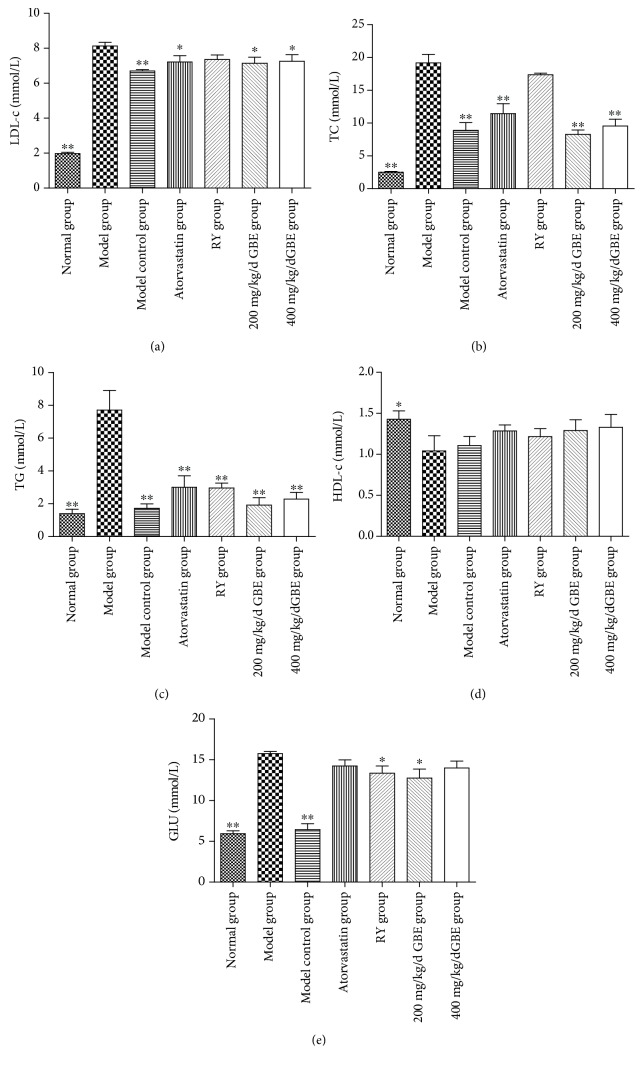
Effects of GBE on serum lipid and glucose profiles. ^∗^*P* < 0.05 and ^∗∗^*P* < 0.01, compared to the model group. GBE: ginkgo biloba leaf extract (normal group *n* = 18, model group *n* = 7, model control group *n* = 7, atorvastatin group *n* = 11, rapamycin group *n* = 15, 200 mg/kg/day GBE group *n* = 10, and 400 mg/kg/day GBE group *n* = 12).

## References

[1] Tian J., Popal M. S., Liu Y. (2019). Ginkgo biloba leaf extract attenuates atherosclerosis in streptozotocin-induced diabetic ApoE-/- mice by inhibiting endoplasmic reticulum stress via restoration of autophagy through the mTOR signaling pathway. *Oxidative Medicine and Cellular Longevity*.

